# Multi-Parametric Optimization of 3D-Printed Components

**DOI:** 10.3390/polym17010027

**Published:** 2024-12-26

**Authors:** Calin Vaida, Grigore Pop, Paul Tucan, Bogdan Gherman, Doina Pisla

**Affiliations:** 1 CESTER-Research Center for Industrial Robots Simulation and Testing, Technical University of Cluj-Napoca, 400114 Cluj-Napoca, Romania; calin.vaida@mep.utcluj.ro (C.V.); bogdan.gherman@mep.utcluj.ro (B.G.); doina.pisla@mep.utcluj.ro (D.P.); 2 Dassault Systèmes Solutions Center, Technical University of Cluj-Napoca, 400114 Cluj-Napoca, Romania; 3 Technical Sciences Academy of Romania, 26 Dacia Bvd, 030167 Bucharest, Romania

**Keywords:** 3D printing accuracy, FDM printing, 3D printing process parameters

## Abstract

This study explores the experimental and theoretical optimization of process parameters to improve the quality of 3D-printed parts produced using the Fused Deposition Modeling technique. To ensure the cost-effective production of high-quality components, advancements in printing strategies are essential. This research identifies optimal 3D printing strategies to enhance the quality of finished products. Form and dimensional tolerances were assessed using a 3D Coordinate Measuring Machine, and the resulting data were analyzed via Design Expert software version 9.0.6.2. Design Expert for experimental design was utilized and an Analysis of Variance was conducted to validate the models’ accuracy. The results indicate that a 45° raster angle, combined with internal raster values between 0.5048 and 0.726, minimizes flatness, cylindricity, and dimensional deviations by optimizing deposition patterns and thermal dynamics. Internal raster values below 0.308 resulted in insufficient support and greater deviations, while higher values enhanced stability through improved interlayer adhesion. Experimental validation confirmed these parameter settings as optimal for producing precise and consistent 3D-printed parts.

## 1. Introduction

Additive manufacturing, commonly known as 3D printing, emerged as a transformative technology with significant implications across various scientific disciplines. By creating 3D objects layer-by-layer from digital models, 3D printing enables the fabrication of complex structures with exceptional precision and minimal waste. This technology contrasts with traditional manufacturing methods, which often involve cutting or molding and often generate excess material or scrap. The technology’s ability to facilitate rapid prototyping, customization, and the fabrication of intricate geometries has led to widespread adoption across industrial and research sectors [1,2,3,4].

Among the various 3D printing methods, Fused Deposition Modeling (FDM) is the most widely used due to its cost-effectiveness and accessibility. This technique involves the extrusion of thermoplastic materials through a heated nozzle that moves according to a design, building the object layer-by-layer. FDM is particularly-suited for prototype development and production of functional parts [1,2,3,4]. Other advanced techniques include Stereolithography (SLA), which uses ultraviolet light to cure liquid resin into high-resolution solid layers, and Selective Laser Sintering (SLS), which employs lasers to fuse powdered materials into strong and durable objects [5,6,7,8,9]. These methods address distinct needs across industries, ranging from creating biomedical models to fabricating components for aerospace and automotive applications.

3D printing is playing an increasingly important role in various fields of scientific research. In biomedical engineering, it enables the creation of patient-specific models for surgical planning, custom prosthetics, and even bio-scaffolds for tissue engineering. The technology’s precision allows for the fabrication of complex organic structures that replicate biological tissues, paving the way for advancements in regenerative medicine [10,11,12,13]. In material science, 3D printing facilitates the development of novel materials and structures with customized properties. Researchers are investigating the use of functionalized materials, such as conductive polymers and metallic composites, which can be printed into structures with unique mechanical, electrical, or thermal characteristics. This innovation is expanding possibilities for creating advanced materials used in electronics, energy storage and sensing technology [14,15]. In environmental science, 3D printing promotes sustainable manufacturing by minimizing material waste and enabling localized production. The ability to rapidly prototype and test new designs also accelerates progress in environmental technologies, such as water purification systems and renewable energy devices [16].

In medical rehabilitation, the precision of 3D printing is instrumental in developing personalized devices tailored to the unique needs of patients with neuromotor disabilities. By aligning with patient-specific anatomical data, 3D-printed rehabilitation devices offer affordable, customizable solutions for home-based therapy. These applications are particularly beneficial for individuals with conditions such as stroke, Parkinson’s disease, multiple sclerosis, and spinal cord injuries, where tailored devices enhance motor retraining and significantly improve quality of life [17,18,19,20].

Dimensional accuracy is critical for the functionality and reliability of 3D-printed parts. Precise dimensions are essential for proper fit and assembly, particularly in industries such as healthcare, aerospace, and automotives. In medical applications, for instance, even minor deviations can compromise safety and performance, emphasizing the importance of high precision [21,22,23,24]. Studies have highlighted several factors influencing accuracy, including material selection, printing parameters, and printer resolution. While higher resolution printers yield superior accuracy, they often come with increased costs and longer production times [25,26,27]. Additionally, quality control measures are essential to minimize common 3D printing defects such as warping, layer misalignment, and shrinkage, which can adversely affect dimensional fidelity and mechanical properties [28].

This study focuses on optimizing raster angles and enhancing internal raster structures to improve dimensional accuracy in FDM. Variations in these parameters can lead to dimensional deviations in printed parts, thus compromising their performance and reliability. By refining raster strategies and internal patterns, the research aims to minimize errors such as warping and anisotropic behavior while preserving mechanical strength. These advancements are crucial for producing high-quality components in applications requiring tight tolerances, such as the aerospace, automotive, and biomedical fields.

The findings of this study aim to contribute to the development of more accurate and efficient additive manufacturing techniques. By addressing challenges related to precision and material deposition uniformity, the research seeks to enhance the reliability of 3D printing for both fundamental research and practical applications. As the demand for individualized rehabilitation devices increases, the ability to produce cost-effective, high-precision components is essential for expanding access to such technologies [26,27,28].

This paper is structured as follows: Section 2 presents the materials and methods, describing the experimental setup and evaluation techniques used to assess accuracy. Section 3 details the results, while Section 4 and Section 5 provide the discussions and conclusions, respectively.

## 2. Materials and Methods

Statistical modeling for the experimental evaluation of raster angles and enhanced internal raster structures to improve the dimensional accuracy of 3D printed parts was conducted using Design Expert (Minneapolis, MN, USA) [29] software. This approach involves applying statistical techniques to model and analyze the performance of the 3D printing process of the Fortus 380mc printer from Stratasys (Minneapolis, MN, USA) [30]. The aim of this research was to model and understand the behavior of the 3D printing process. Statistical modeling was utilized to characterize the relationships between process parameters and the quality of the printed parts, allowing for the identification of key factors that influence the printing process and helps optimizing it to achieve desired outcomes. Additionally, mathematical optimization techniques were used to find the best possible solution within a defined set of constraints [31,32]. A detailed experimental setup is provided in the following section.

### 2.1. Methodology and Programing of the Experiments

Design Expert software is used facilitate the creation and execution of experimental designs such as determining the optimum formula for a given preparation. In addition to optimization, the software provides tools to analyze and interpret the factors influencing the experiment. Design Expert offers three distinct research directions depending on the experimental design to be carried out. These are screening, characterization, and optimization [33].

The first action in the assessment process is to create the 3D design using Siemens NX [34]. The design is then converted into an STL file, the standard format for 3D printing. Once the STL file is generated, process parameters (such as raster angle, layer height, and temperature) are defined for the experiment. These input variables are used to design experiments using the Design of Experiments (DOEs) method, which facilitates systematic testing and analysis of various parameter combinations. The design matrix generated during the DOE setup serves as the foundation for the 3D printing process, yielding a range of printed samples. Once the printing is complete, the printed parts are measured to assess their dimensional accuracy and other properties. The measurement data collected are analyzed, and modeling techniques are applied to understand the effects of different parameters. Based on the analysis, the process parameters are optimized to improve the overall quality and performance of the 3D-printed components.

The flowchart illustrates a structured approach to optimizing 3D printing processes by leveraging CAD design, experimental programming (DOE), and analysis techniques to achieve refined and improve outcomes.

### 2.2. Experimental Work

In order to determine the number of samples required for the experimental trials, the parameters of the 3D printer were analyzed. The printer used was the Fortus 380mc 3D printer from the Stratasys Fortus series, known for its high-performance Fused Deposition Modeling (FDM) capabilities. The standard deviation of dimensional accuracy of the Fortus 380mc varies depending on the material, specific print parameters, and geometric complexity of the printed part. While no universal standard deviation applies to all prints, studies and technical reports provide useful insights. For the Fortus series, including the 380mc, typical dimensional tolerances are ±0.127 mm or ±0.0015 mm per mm of the part, whichever is greater. These tolerances are achieved under controlled conditions using standard materials such as ABS or ASA. Several factors can affect this dimensional accuracy:

Material: Different materials (e.g., ABS, polycarbonate, ULTEM) have varying shrinkage rates and mechanical properties, which affect the accuracy of printed part.

Part Size and Geometry: Larger or more complex parts generally exhibit more variation due to warping, internal stresses, or layer bonding issues.

Layer Height and Speed: A finer layer height (e.g., 0.127 mm) typically results in more accurate parts compared to thicker layers (e.g., 0.254 mm), though they require longer print time.

Post-Processing: Factors such as cooling rate, support removal, and finishing processes can introduce slight deviations from the printed dimensions.

Based on various industry reports and internal testing, the standard deviation of dimensional accuracy for Fortus printers typically falls within the range of ±0.05 mm to ±0.02 mm for well-calibrated systems with optimal settings.

These figures suggest that the dimensional variation between printed parts typically falls within this range, depending on the complexity and size of the part. For more precise results, specific user testing and calibration for each print job are often required.

Considering an estimated standard deviation of 0.06 mm and a marginal error of 0.02 mm, the required number of samples can be determined using Equation (1). In this equation, *n* is the required sample size, *Z* is the *Z*-score for the 95% confidence level (1.96), *s* is the standard deviation of the population or sample, and *E* is the desired margin of error. By substituting the above values into Equation (1) yields a required samples size of 34.5. Since there are 13 configurations, the sample size is increased to 39, resulting in 3 samples for each configuration.

(1) $$n={\left(\frac{Z\cdot s}{E}\right)}^{2}$$

The material used for the experiments is Acrylonitrile Butadiene Styrene -ABS-M30 with the properties detailed in Table 1.

Toolpath Parameters Window contains detailed options for customizing the print toolpath. The possible settings include the following:

- Fill Style: Options for the fill pattern used in the printed part, such as “One contour/rasters” and specifying internal styles like “Solid”.
- Contours: Adjustments for contour width and the number of contours.
- Additional Settings: Options for shrink factors and drink factors (typically used to compensate for material shrinkage).
- Enhanced Surfaces: Control for surface raster styles and widths, with options for both visible and internal rasters.
- Raster Fill: Allows customization of raster width and angle.
- Sparse Fill: Settings related to sparse fill structures, often used to reduce material usage without compromising part strength.

This setup provides highly customizable control over the 3D printing process, including the density of the part, surface finish quality, and internal structure.

**Figure 1:**
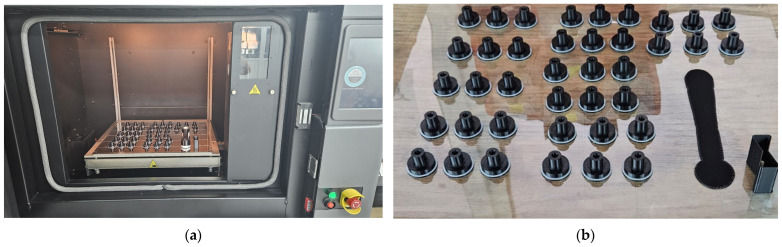
Experimental setup: (**a**) Fortus 380mc 3D printer, (**b**) printed parts.

Based on the calculated statistical data, the 39 samples were printed on a single foil, to minimize variability in printing parameters and material usage. The experimental printing and the final results are illustrated in Figure 1.

### 2.3. Measurements

For dimensional and form deviations, the Axiom Too 3D measuring machine, a coordinate measuring machine (CMM) produced by Aberlink (Aberlink Ltd., Gloucestershire, UK), was used. This CMM is designed for precision measurement in various industrial applications including dimensional inspection, quality control, and reverse engineering. For each unit (plane, cylinder) a minimum of 15 points evenly distributed were measured across 3 different cross sections and the results were documented in the report. The following parameters were measured:

- Form deviations, including flatness and cylindricity;
- Dimensional tolerances, showing the actual deviation from the nominal size.

The measurements were conducted in the 3D measurement laboratory of the Technical University of Cluj-Napoca, under controlled conditions at 20 °C and 50% humidity, using a Axiom too CMM (Gloucestershire, UK), CMM and its firmware software (Aberlink 3D, Version 4.22).

**Figure 2:**
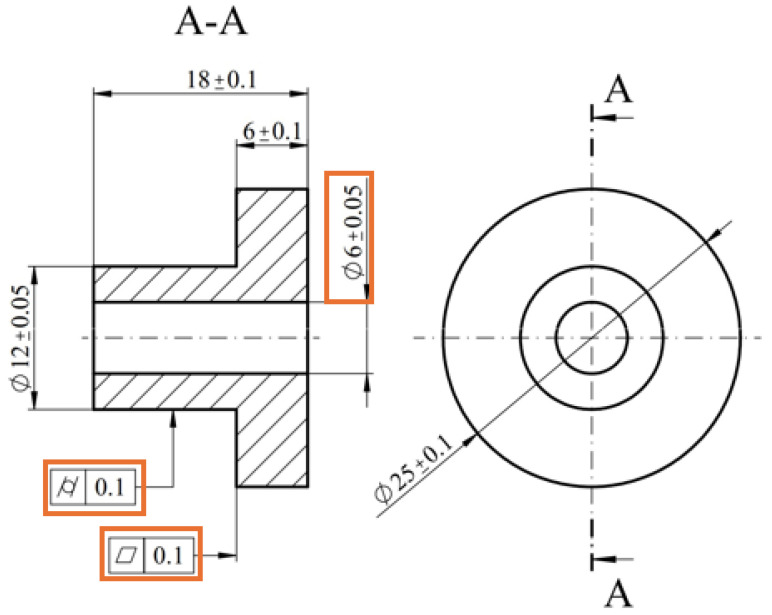
2D drawing of the printed part.

Figure 2 illustrates the drawing of the 3D-printed part, where the highlighted dimensions represent the target dimensions, measured during the experimental tests.

### 2.4. Experimental Modeling

The factors analyzed for their influence on the overall geometrical shape of the 3D printed parts are presented in Table 2. The design matrix, generated by the DOE software version 9.0.6.2, was created after establishing the lower and upper limits of the process parameters.

After the measurements were performed, the average values of the flatness, cylindricity and dimensional deviations were entered to in the design matrix. The results were then analyzed using Design Expert software.

## 3. Results

After performing the ANOVA analysis, the mathematical equations in terms of the actual factors was determined as follows: measured flatness (Equation (2)), measured cylindricity (Equation (3)), measured deviation from the nominal size of the 6 mm hole (Equation (4))

(2) $$nf=0.41965-1.06916\cdot A-1.32746\cdot 10^{-3}\cdot B+1.13606\;\cdot 10^{-3}\cdot A\cdot B+0.83555\cdot A^{2}+7.59766\cdot 10^{-6}\cdot B^{2}$$

(3) $$nc=0.068379-0.11868\cdot A+5.0341\cdot 10^{-5}\cdot B-6.37397\cdot 10^{-4}\cdot A\cdot B+0.12654\cdot A^{2}+7.59776\cdot 10^{-6}\cdot B^{2}$$

(4) $$nd=5.06443-33.39697\cdot A-1.43143\cdot 10^{-3}\cdot B+0.013982\cdot A\cdot B+68.07322\cdot A^{2}-3.03978\cdot 10^{-5}\cdot B2-0.023423\cdot A^{2}\cdot B+8.07163\cdot 10^{-5}\cdot A\cdot B^{2}-43.35152\cdot A^{3}-4.05946\cdot 10^{-8}\cdot B^{3}$$

where: *A* represents the enhanced internal rasters, and *B* represents the raster angle.

**Figure 3:**
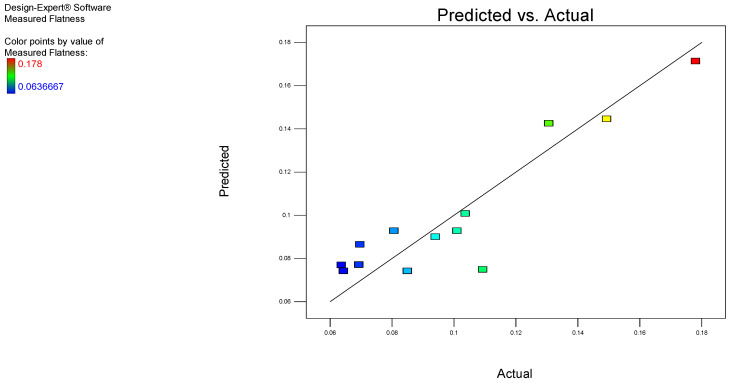
Correlation chart for flatness.

Furthermore, the experimental data and the predicted values based on the aforementioned model are plotted in Figure 3. The results indicate a strong correlation, as evidenced by the determination factor, R^2^.

The adequacy of the developed model was subsequently checked using the ANOVA method, and the quadratic model was deemed appropriate. The coefficient R^2^ = 0.8384, indicates that the model is significant. Predicted R^2^ is a measure of how well the model predicts the response value. The obtained predicted R^2^ value of 0.5672 is reasonably close to the adjusted R^2^ value of 0.7229, with a difference of less than 0.2.

An adequate or sufficient precision value greater than 4 indicates that the model is estimating the response function adequately. This is defined as the signal-to-noise ratio, which compares the range of predicted values to their average standard error at the design stage. The calculated adequate precision value of 7.770 indicates an adequate signal-to-noise ratio. In conclusion the developed model is suitable for navigating the design space.

**Figure 4:**
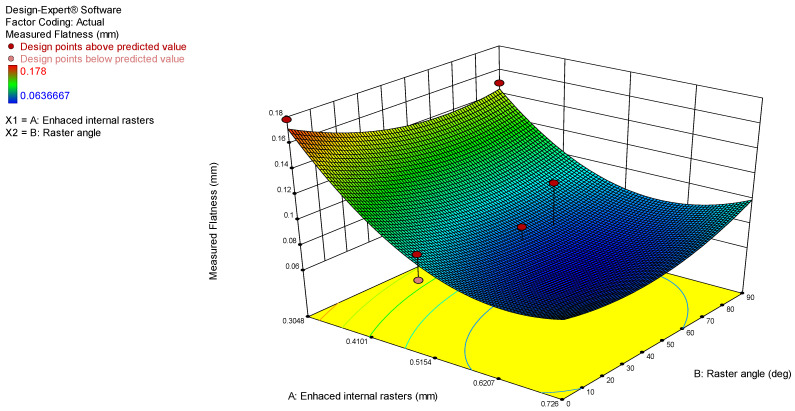
3D surface model graph for flatness.

**Figure 5:**
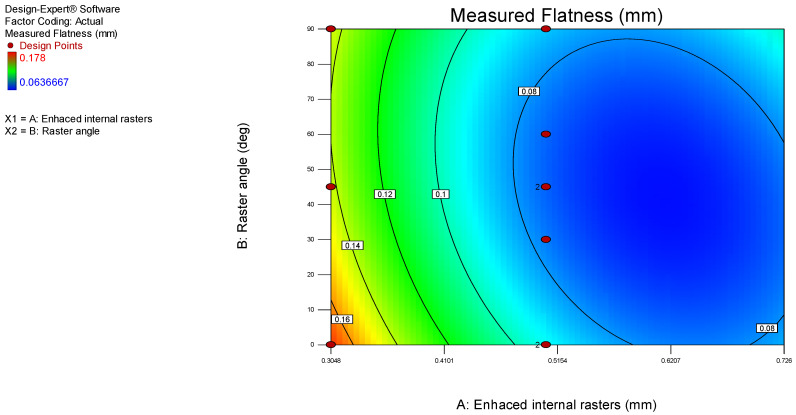
Contour model graph for flatness.

The model graphs presented in Figure 4 and Figure 5 show the influence of the analyzed process parameters on the flatness deviation values. The lowest measured flatness value of 0.5048 was obtained with enhanced internal rasters. However, the enhanced internal rasters decrease, the flatness value increases to 0.16 mm. Additionally, the flatness deviation increases from 0.64 to 0.1 mm as the raster angle rises from 0 to 90 degrees.

**Figure 6:**
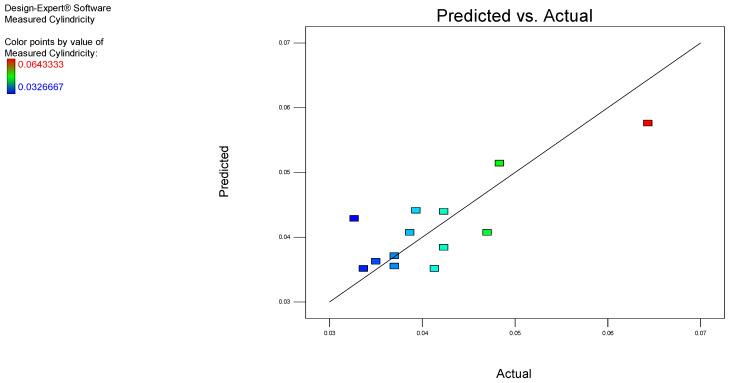
Correlation chart for cylindricity.

Figure 6 indicates both the experimental and the predicted data for cylindricity using the aforementioned model, indicating a strong correlation.

The obtained predicted R^2^ value of 0.6547 suggests that the model is suitable for navigating the design space. The adequate precision for this model was 5.152, indicating an adequate signal. Additionally, the chart demonstrates a strong correlation between the predicted and the experimental values.

**Figure 7:**
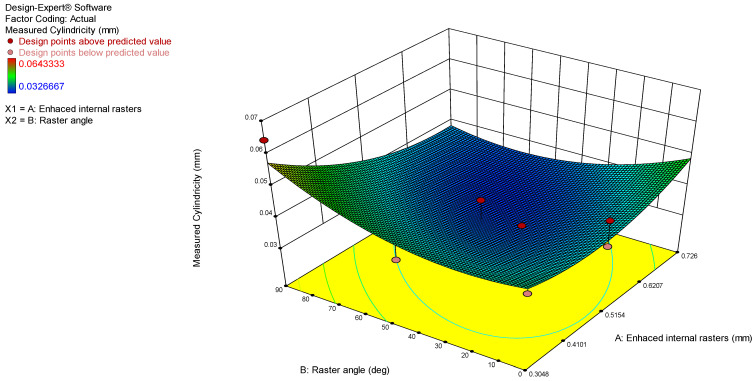
3D surface model graph for cylindricity.

**Figure 8:**
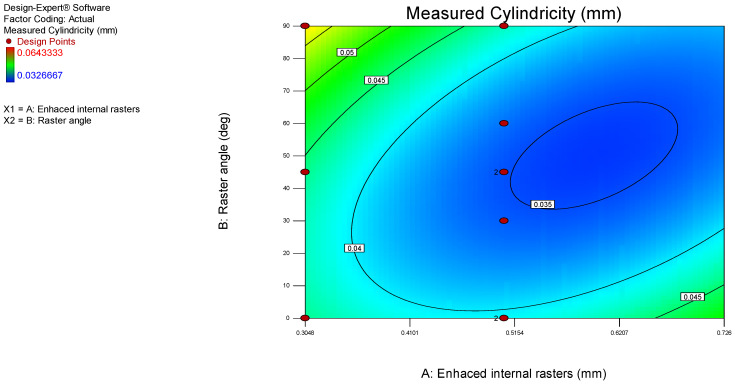
Contour model graph for cylindricity.

Furthermore, the 3D surface model and contour graphs were plotted to identify the influence of process parameters on the obtained cylindricity values, as presented in Figure 7 and Figure 8. The lowest measured cylindricity value of 0.5048 was obtained with enhanced internal rasters. The cylindricity deviation remains unaffected by the increase in raster angle from 0 to 90 degrees when the enhanced internal rasters are kept constant at 0.5 mm.

**Figure 9:**
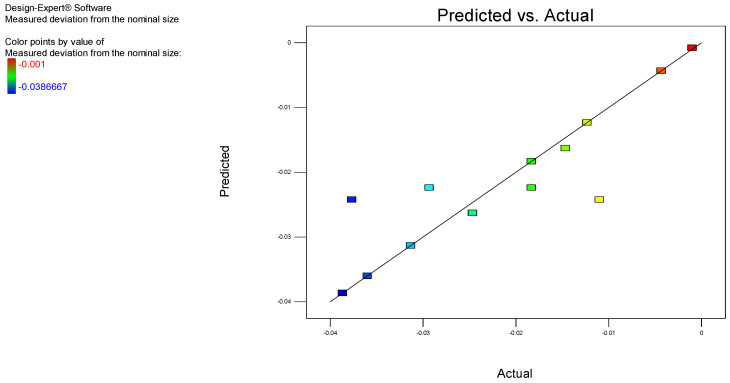
Correlation chart for the measured deviation.

**Figure 10:**
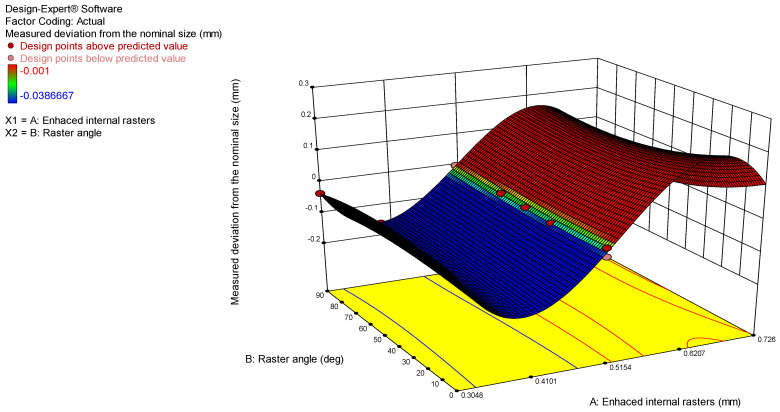
3D surface model graph for the measured deviation.

**Figure 11:**
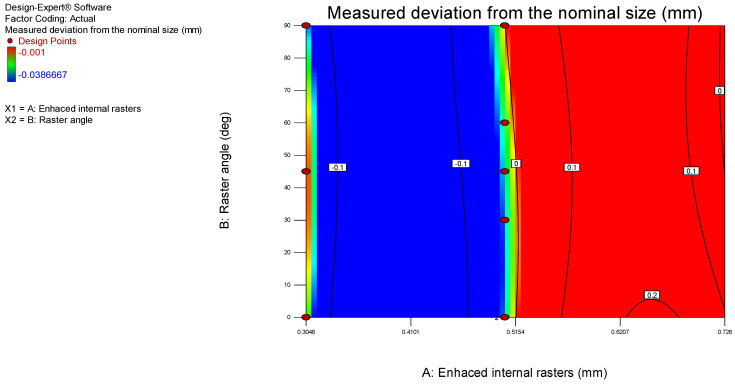
Contour model graph for the measured deviation.

The correlation between the experimental and predicted values is presented in Figure 9. The predicted R^2^ value of 0.7772 indicates that the model is suitable for navigating the design space. Additionally, the 3D surface model and contour graphs were plotted and presented in Figure 10 and Figure 11 to analyze the influence of process parameters on the actual deviation from the nominal size. The best accuracy was achieved when printing with enhanced internal rasters of 0.5048. The actual deviation increases as this parameter either decreases or increases.

### Results Validation

**Figure 12:**
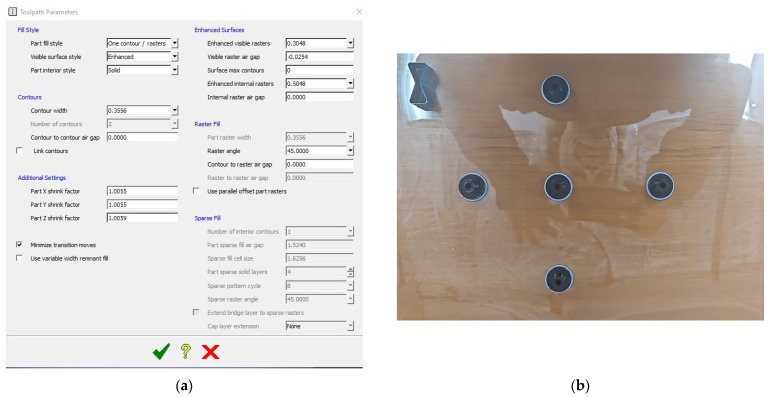
Experimental setup: (**a**). Process parameter input in Fortus 380. (**b**). Five printed samples on a single foil.

Based on the analysis results, 5 pieces were printed with the internal rasters set at 0.5048 and the raster angle configured at 45°. The setup and a sample are presented in Figure 12 and the measured results are detailed in Table 3.

The validation printing yielded measured values that aligned closely with the predictions of the mathematical model. Specifically, the measured flatness values were below 0.05 mm, cylindricity did not exceed 0.041 mm, and the actual deviation from the nominal size was less than 0.045 mm.

## 4. Discussion

The deviations observed in flatness and cylindricity measurements provide valuable insights into the effect of print parameters such as raster angle and enhanced internal rasters on the 3D printing process. Flatness, a critical geometric feature, tends to vary substantially depending on these key parameters. In this study, the maximum deviations for flatness occurred when printing with a raster angle of 0° and an enhanced internal raster value of 0.3048. This is an expected outcome because a 0° raster angle typically aligns toolpaths in one direction, leading to non-uniform material deposition. This uneven deposition ultimately affects the surface smoothness and flatness. The non-uniform cooling and possible warping are more pronounced at this raster angle, resulting in higher deviations. In contrast, when the raster angle was set to 45° and the enhanced internal rasters value increased to 0.5048, flatness deviations improved significantly. This configuration likely promotes a more balanced material deposition and even cooling, thereby minimizing distortions in the part’s flat surface.

A noteworthy observation is that increasing the enhanced internal raster value from 0.5048 to 0.726 maintained flatness deviations below 0.1 mm, irrespective of the raster angle, which ranged from 0° to 90°. This suggests that once the raster settings reach a certain threshold, the internal raster structure becomes more robust, providing enhanced support for the external surfaces and a corresponding reduction in deviations. This behavior may be attributed to improved interlayer adhesion at increased raster values, which prevents surface irregularities during the cooling process. Moreover, it highlights that while raster angles play a significant role, the impact of enhanced internal rasters may become the dominant factor in controlling flatness as the values approach higher levels.

When examining cylindricity, the results similarly indicate that the optimal printing performance is achieved with a raster angle of 45° and enhanced internal rasters values between 0.5048 and 0.726. This configuration of settings seems to facilitate a more uniform cylindrical shape, likely due to balanced forces acting on the part during the printing process. The inherent circular symmetry of cylinders might benefit more from these mid-range raster values, as they provide adequate internal support while minimizing the risk of layer shifts or distortions in the cylindrical shape.

However, the highest cylindricity deviations were recorded when printing at a lower enhanced internal raster value of 0.308 while maintaining a raster angle of 45°. These increased deviations can be attributed to insufficient internal structure support, which might allow for minor shifts or distortions in the cylindrical features. Such distortions may result from inconsistencies in material deposition or thermal expansion during the printing process. At lower enhanced internal raster values, the internal support may lack the necessary robustness to counteract these distortions, resulting in greater cylindricity deviations.

Regarding the dimensional accuracy, the best results were achieved when printing parts with an enhanced internal raster value of 0.5048 and a raster angle of 45°. These settings likely provide an optimal balance between internal support and external surface smoothness, minimizing deviations from the nominal size. Dimensional accuracy is a critical metric, especially in applications where the final part must meet tight tolerances. The results demonstrate that deviations from the nominal size tend to increase both when the value of enhanced internal rasters exceeds 0.05154 or decreases below 0.03048. This non-linear relationship indicates that there is a narrow range of enhanced internal raster values that yield the best results for dimensional accuracy. If the raster values are too low, the part may lack sufficient internal stability, leading to shrinkage or warping. Conversely, if the raster values are too high, overcompensation in the internal structure could lead to expansion or bulging, which would also negatively impact dimensional accuracy.

This study reveals that the accuracy of FDM printing is significantly influenced by the interplay of raster angle and enhanced internal raster values, which govern material deposition, cooling, and structural support. Flatness deviations peaked at 0° raster angle combined with low internal raster values (0.3048) due to uneven deposition and cooling, causing warping. Optimal flatness, cylindricity, and dimensional accuracy were achieved at a 45° raster angle and internal raster values between 0.5048 and 0.726. These settings provided balanced deposition patterns, thermal dynamics, and internal structure stability. Cylindricity improved under these conditions due to the uniform distribution of forces during the process. Low internal raster values (<0.308) provided insufficient support, increasing distortions, while higher values (>0.5048) enhanced interlayer adhesion, thus stabilizing the parts. Experimental results validated these settings as optimal for minimizing geometric deviations.

The theoretical findings were validated experimentally, as a set of five parts printed using the values identified through the mathematical model were all within the predicted deviation limits.

## 5. Conclusions

The analysis of dimensional and geometrical shape accuracy in 3D printing highlights the importance of applying specific constraints when selecting machining parameters to ensure optimal precision. This study revealed that process parameters, such as the raster angle and enhanced internal rasters, significantly influence the final accuracy of printed parts. Among the parameters tested, the most accurate parts were consistently produced with a raster angle of 45° and an enhanced internal raster value of 0.5048. This combination minimized deviations in flatness, cylindricity, and dimensional accuracy, suggesting that these settings provide a balanced internal structure that effectively supports the external geometry of the parts. Careful calibration of these parameters clearly improve print quality by minimizing the variations caused by thermal expansion, material shrinkage, or inconsistencies in layer deposition. Optimizing these key parameters enables manufacturers to achieve higher precision in their printed components, making this approach ideal for applications where tight tolerances and superior surface finishes are critical. However, further investigations are recommended to explore the interplay of additional factors, such as print speed, material types, and layer thickness. Exploring these variables could further refine the process and allow for improved control over part accuracy in complex geometries.

## Figures and Tables

**Table 1:** Material properties.

Characteristic	Value
Tensile Strength (XZ)	~32 MPa
Flexural Strength (XZ)	~61 MPa
Elongation at Break (XZ)	6%
Heat Deflection Temperature (at 0.45 MPa)	96 °C
Layer Thickness	0.254–0.330 mm

**Table 2:** Design matrix for the measured parameters.

	Input Parameters		Responses		
Run	Enhanced Internal Raster [mm]	Raster Angle [deg]	Measured Flatness [mm]	Measured Cylindricity [mm]	Actual Deviation from the Nominal Size [mm]
1	0.729	90	0.104	0.042	−0.031
2	0.5048	0	0.081	0.039	−0.011
3	0.762	0	0.094	0.048	−0.039
4	0.729	45	0.069	0.037	−0.012
5	0.5048	30	0.064	0.037	−0.025
6	0.3048	90	0.149	0.064	−0.036
7	0.5048	45	0.085	0.034	−0.029
8	0.5048	60	0.109	0.035	−0.015
9	0.3048	0	0.178	0.042	−0.018
10	0.3048	45	0.131	0.039	−0.004
11	0.5048	90	0.070	0.033	−0.001
12	0.5048	0	0.101	0.047	−0.038
13	0.5048	45	0.064	0.041	−0.018

**Table 3:** Measured values.

Part No.	Enhanced Internal Raster [mm]	Raster Angle [deg]	Measured Flatness [mm]	Measured Cylindricity [mm]	Actual Deviation from the Nominal Size [mm]
1	0.5048	45	0.039	0.032	−0.018
2	0.5048	45	0.038	0.031	−0.031
3	0.5048	45	0.050	0.041	−0.038
4	0.5048	45	0.041	0.020	−0.039
5	0.5048	45	0.046	0.032	−0.044

## Data Availability

The original contributions presented in this study are included in the article. Further inquiries can be directed to the corresponding authors.
